# Efficacy and safety of lipegfilgrastim versus pegfilgrastim in elderly patients with aggressive B cell non-Hodgkin lymphoma (B-NHL): results of the randomized, open-label, non-inferiority AVOID neutropenia study

**DOI:** 10.1007/s00520-020-05711-7

**Published:** 2020-09-17

**Authors:** Hartmut Link, G. Illerhaus, U. M. Martens, A. Salar, R. Depenbusch, A. Köhler, M. Engelhardt, S. Mahlmann, M. Zaiss, A. Lammerich, P. Bias, A. Buchner

**Affiliations:** 1 Private Practice Hematology Medical Oncology, Finkenhain 8, 67661 Kaiserslautern, Germany; 2 Hematology, Oncology and Palliative Medicine Clinic, Klinikum Stuttgart, Stuttgart, Germany; 3 Hematology, Oncology and Palliative Medicine Clinic, SLK-Clinics, Heilbronn, Germany; 4 Hospital del Mar Paseo Marítimo, Hematology, Barcelona, Spain; 5 Oncology Practice, Gütersloh, Gütersloh, Germany; 6 Hematology and Oncology Collective Practice, Asklepios Clinic Specialist Medical Centre Langen, Langen, Germany; 7 Internal Medicine Clinic I, Hematology, Oncology and Stem Cell Transplantation, University Clinic, Faculty of Freiburg, Freiburg, Germany; 8 Hematology/Oncology and Nephrology Clinic, Friedrich-Ebert-Hospital Neumünster, Neumünster, Germany; 9 Interdiscliplinary Practice for Oncology and Hematology, Freiburg, Germany; 10 Teva Pharmaceuticals Industries, Ulm, Germany

**Keywords:** Lipegfilgrastim, Pegfilgrastim, Granulocyte colony, stimulating factor (G-CSF), Chemotherapy-induced neutropenia, Febrile neutropenia, B cell non-Hodgkin lymphoma

## Abstract

**Background:**

Lipegfilgrastim has been shown to be non-inferior to pegfilgrastim for reduction of the duration of severe neutropenia (DSN) in breast cancer patients. This open-label, non-inferiority study assessed the efficacy and safety of lipegfilgrastim versus pegfilgrastim in elderly patients with aggressive B cell non-Hodgkin lymphoma (NHL) at high risk for chemotherapy-induced neutropenia.

**Patient and methods:**

One hundred and one patients (median age, 75 years) were randomized to lipegfilgrastim or pegfilgrastim (6 mg/cycle) during six cycles of R-CHOP21.

**Results:**

Lipegfilgrastim was non-inferior to pegfilgrastim for the primary efficacy endpoint, reduction of DSN in cycle 1. In the per-protocol population, mean (standard deviation) DSN was 0.8 (0.92) and 0.9 (1.11) days in the two groups, respectively; the adjusted mean difference between groups was − 0.3 days (95% confidence interval, − 0.70 to 0.19). Non-inferiority was also demonstrated in the intent-to-treat population. The incidence of severe neutropenia in cycle 1 was 51% (21/41) in the lipegfilgrastim group and 52% (23/44) in the pegfilgrastim group. Very severe neutropenia (ANC < 0.1 × 10^9^/L) in cycle 1 was reported by 5 (12%) patients in the lipegfilgrastim group and 8 (18%) patients in the pegfilgrastim group. However, over all cycles, febrile neutropenia (strict definition) was reported by only 1 (2%) patient in each treatment group (during cycle 1 in the lipegfilgrastim group and cycle 6 in the pegfilgrastim group). The mean time to absolute neutrophil count recovery (defined as ≥ 2.0 × 10^9^/L) was 8.3 and 9.4 days in the two groups, respectively. Serious adverse events occurred in 46% of patients in each group; none were considered treatment-related. Eight patients died during the study (2 in the lipegfilgrastim group, 5 in the pegfilgrastim group, and 1 who died before starting study treatment). No deaths occurred during the treatment period, and all were considered to be related to the underlying disease.

**Conclusions:**

This study shows lipegfilgrastim to be non-inferior to pegfilgrastim for the reduction of DSN in elderly patients with aggressive B cell NHL receiving myelosuppressive chemotherapy, with a comparable safety profile.

**Trial registration number:**

ClinicalTrials.gov identifier NCT02044276; EudraCT number 2013-001284-23

**Electronic supplementary material:**

The online version of this article (10.1007/s00520-020-05711-7) contains supplementary material, which is available to authorized users.

## Introduction

Elderly patients with non-Hodgkin lymphoma (NHL) receiving chemotherapy, such as R-CHOP, are at high risk of developing clinically significant neutropenia [1–3], which can lead to dose reductions, cycle delays, or even treatment discontinuation. Clinically significant neutropenia is defined as grade 4 neutropenia (absolute neutrophil count [ANC] < 0.5 × 10^9^/L) according to the Common Terminology Criteria for Adverse Events, the most widely used scale for grading chemotherapy-related cytopenias [4]. Maintenance of chemotherapy intensity is important in patients with NHL, as there is strong evidence that survival is negatively impacted by reductions in relative dose intensity in this population [5–8]. Use of recombinant granulocyte colony–stimulating factors (G-CSFs) is recommended for patients at high risk of chemotherapy-induced neutropenia [9–11], and has been shown to improve survival, especially in elderly patients and those receiving dose-dense regimens [5].

Short-acting G-CSFs, such as filgrastim (Neupogen®; Amgen Inc., Thousand Oaks, CA, USA), require daily subcutaneous injections during each chemotherapy cycle. Pegylation decreases plasma clearance of filgrastim and extends its half-life in the body, allowing for less frequent dosing. Lipegfilgrastim (Lonquex®; Teva B.V., Haarlem, Netherlands) is a long-acting G-CSF indicated for reduction of the duration of neutropenia and the incidence of febrile neutropenia (FN) in adult patients receiving cytotoxic chemotherapy [12]. Lipegfilgrastim is glycopegylated in a site-specific manner, resulting in greater structural homogeneity and improved pharmacokinetic and pharmacodynamic properties compared with conventionally pegylated G-CSFs [13, 14]. Lipegfilgrastim has been shown to induce a longer-lasting increase in ANC than an equivalent dose of the conventionally glycopegalated long-acting G-CSF, pegfilgrastim (Neulasta®; Amgen Inc., Thousand Oaks, CA, USA) [15]. This may reflect the higher cumulative exposure and slower clearance of lipegfilgrastim compared with pegfilgrastim [15]. Lipegfilgrastim, administered once per chemotherapy cycle, has been shown to be non-inferior to pegfilgrastim with respect to duration of severe neutropenia (DSN, defined as the number of days with grade 4 neutropenia [ANC < 0.5 × 10^9^/L]) in breast cancer patients [16].

Pegfilgrastim has been shown to be effective for the reduction of DSN and complications of neutropenia in patients with lymphoma receiving chemotherapy regimens associated with a high risk of FN [17–20]. A systematic review undertaken to assess the effectiveness of pegfilgrastim in cancer patients in real-world clinical settings found the risks of FN and FN-related complications to be lower in patients receiving pegfilgrastim than in those receiving short-acting G-CSFs (namely, filgrastim, lenograstim, and biosimilars) [21]. In particular, pegfilgrastim has been shown to be effective for the reduction of DSN in elderly patients with NHL receiving myelosuppressive chemotherapy [22]. This study was undertaken to demonstrate non-inferiority of lipegfilgrastim versus pegfilgrastim in elderly patients with aggressive B cell NHL receiving R-CHOP21, and to compare the efficacy and safety of these long-acting G-CSFs in this elderly NHL population.

## Methods

### Study design and patients

This was a phase 3b, open-label, multicenter study conducted at 31 sites in Germany, Italy, and Spain between March 2014 and December 2017. The study comprised a 2-week screening period, an 18-week, open-label treatment period (6 cycles of R-CHOP21, each of 3 weeks in duration), and a follow-up period of up to 9 months from the start of the first chemotherapy cycle.

Study inclusion and exclusion criteria are summarized in Supplementary Table S1. Patients aged 65–85 years with histologically confirmed aggressive B cell NHL (World Health Organization lymphoma classification criteria [23]) were randomized 1:1 to receive lipegfilgrastim 6 mg or pegfilgrastim 6 mg administered as a single subcutaneous injection on day 3 of each chemotherapy cycle, approximately 24 (± 3) hours after the end of day 2 chemotherapy. During each chemotherapy cycle, patients received (i) rituximab 375 mg/m^2^ intravenously on day 1; (ii) cyclophosphamide 750 mg/m^2^, doxorubicin 50 mg/m^2^, and vincristine 1.4 mg/m^2^ (capped at 2.0 or 1.0 mg) intravenously on day 2; and (iii) prednisone or prednisolone 100 mg orally on days 2 to 6.

The study was approved by independent ethics committees at each study site, and complied with the Declaration of Helsinki, Good Clinical Practice guidelines, and applicable local laws and regulations. Patients provided written informed consent to participate.

### Study assessments

Blood samples were collected for determination of ANC on days 1, 8, and 15 of each cycle, and on days 3, 5, 10, and 12 of cycle 1. ANC analyses were performed by local laboratories. Patients recorded their oral body temperature daily throughout the study (≤ 1 h before chemotherapy administration on days 1 and 2 and before study drug administration on day 3). Patients were also instructed to measure their body temperature if they felt feverish at any time during the day. If body temperature was > 38.0 °C, patients were instructed to measure their body temperature again after 1 h. Patients were instructed to contact study site personnel if their body temperature was > 38.0 °C for more than 1 h.

The primary efficacy measure was ANC, and the primary efficacy outcome was DSN in cycle 1 (number of days with grade 4 neutropenia [ANC < 0.5 × 10^9^/L]). Secondary efficacy measures included the incidence of FN (body temperature > 38.5 °C for ≥1 h and ANC < 0.5 × 10^9^/L [strict definition], or a single body temperature value ≥ 38.3 °C or body temperature ≥ 38.0 °C for ≥1 h and ANC < 1.0 × 10^9^/L [non-strict definition], including cases of neutropenic sepsis or neutropenic serious or life-threatening infection), the incidence of very severe and severe neutropenia during cycle 1 (ANC < 0.1 × 10^9^/L and < 0.5 × 10^9^/L, respectively), the ANC nadir, and the time to ANC recovery (return to ANC ≥ 1.0, ≥ 1.5, and ≥ 2.0 × 10^9^L) in cycle 1. The incidence and severity of infections, rates of hospitalization and intravenous/oral antibiotic administration, and the percentage of chemotherapy dose delivered were also assessed.

Adverse events (AEs) were monitored throughout the study period (classified and graded according to the National Cancer Institute Common Terminology Criteria for Adverse Events, v4.03). Quality of life was assessed prior to administration of chemotherapy in cycles 1 and 4 and at the end of treatment using the European Organisation for Research and Treatment of Cancer Quality of Life Questionnaire C30 (EORTC QLQ-C30) [24] and Functional Assessment of Cancer Therapy-Neutropenia (FACT-N) [25].

### Statistical analysis

Analysis of the primary endpoint was performed for both the per-protocol and intent-to-treat populations. Efficacy data are shown for the per-protocol population unless otherwise noted. Differences in DSN in cycle 1 between treatment groups were analyzed using the two-sided 95% confidence interval (CI), calculated by Poisson regression with identity link, including treatment, body weight, and country as fixed factors and baseline ANC as a covariate. Lipegfilgrastim was considered non-inferior to pegfilgrastim if the upper limit of the two-sided 95% CI for the difference in DSN between groups (lipegfilgrastim minus pegfilgrastim) was < 1 day. A sample size of 50 patients per treatment group provided at least 85% power to reject the null hypothesis.

Secondary endpoints were analyzed by fitting a logistic regression model including the same explanatory variables, and the 95% CI for the odds ratio (lipegfilgrastim versus pegfilgrastim) was calculated. All secondary endpoint analyses were regarded as exploratory; no adjustment for multiple comparisons was performed.

The safety population included all randomized patients who received at least one dose of study medication. All analyses were performed using SAS statistical software version 9.1 or later (SAS Institute, Inc. Cary, NC, USA).

## Results

### Study population

**Fig. 1:**
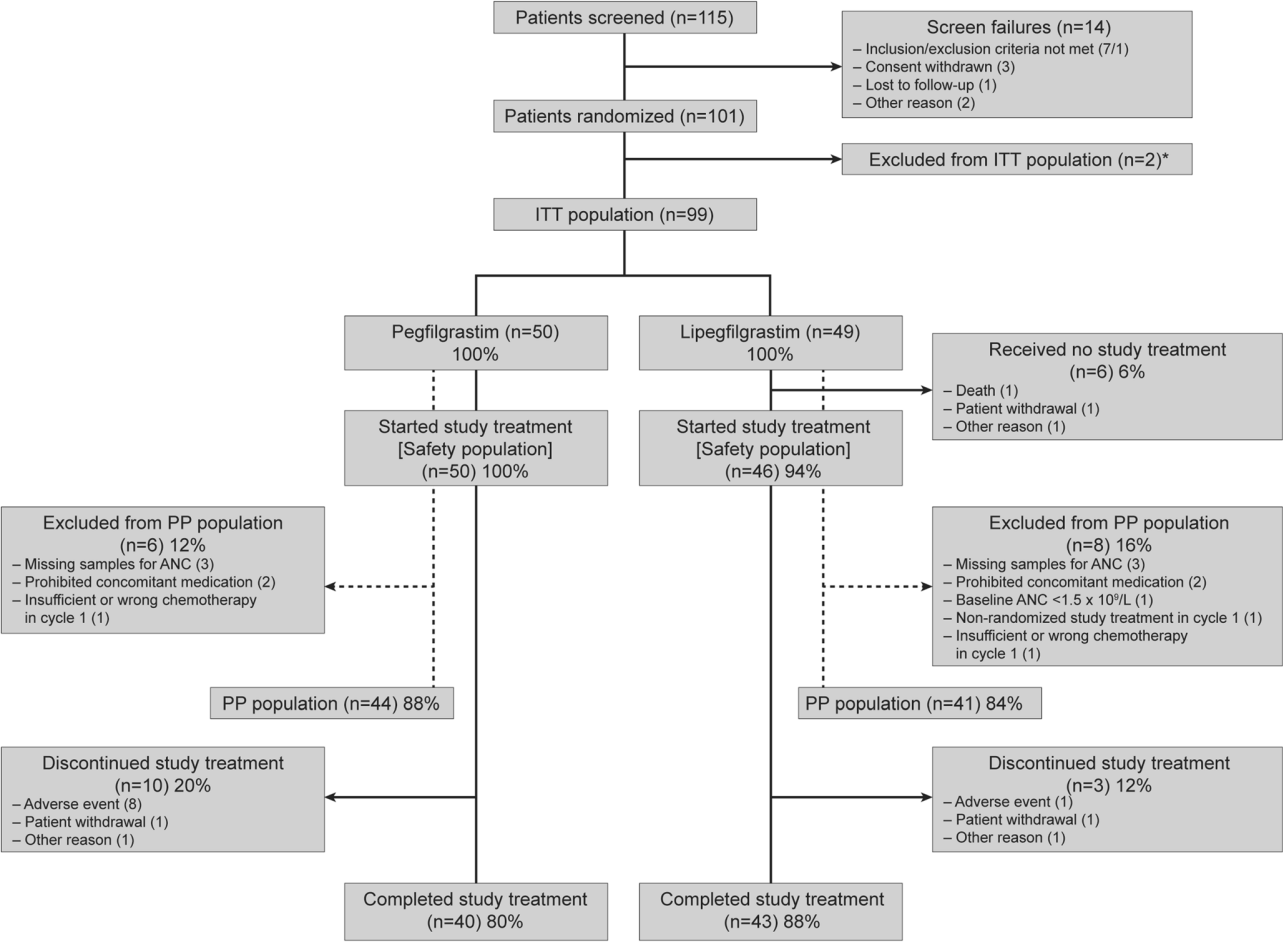
Patient disposition over the study period. *These patients were excluded as they did not receive study drug. ANC absolute neutrophil count, ITT intent-to-treat, PP per-protocol

One hundred and one patients (median age, 75 years [range, 65–82 years]) were enrolled and randomized. Patient disposition is summarized in Fig. 1. The two treatment groups were generally well-matched in terms of patient demographics and baseline disease characteristics (Table 1).

**Table 1:** Patient demographics and baseline disease characteristics (per-protocol population)

Characteristic	Lipegfilgrastim (*N* = 41)	Pegfilgrastim (*N* = 44)
Age (years), *mean* (*SD*)	72.4 (4.65)	75.2 (4.45)
Sex, *n* (%)		
Male	18 (44)	24 (55)
Female	23 (56)	20 (45)
Race, *n* (%)		
White	41 (100)	44 (100)
Time from diagnosis to screening (days), *mean* (*SD*)	40.1 (126.3)	87.9 (350.7)
Type of B cell lymphoma, *n* (%)		
DLBCL	29 (71)	32 (73)
Common morphologic variants	4 (10)	2 (5)
Follicular lymphoma grade IIIb	3 (7)	2 (5)
Other	5 (12)	8 (18)
Stage (modified Ann Arbor), *n* (%)		
I	16 (39)	11 (25)
II	4 (10)	9 (20)
III	9 (22)	12 (27)
IV	11 (27)	12 (27)
Missing	1 (2)	0
Number of sites of extranodal involvement, *mean* (*SD*)	1.6 (2.00)	0.8 (0.82)
B symptoms detected, *n* (%)	14 (34)	12 (27)
IPI score, *mean* (*SD*)	2.2 (1.05)	2.2 (0.94)
ECOG performance status, *n* (%)		
0	26 (63)	22 (50)
1	12 (29)	19 (43)
2	3 (7)	3 (7)

*DLBCL* diffuse large B cell lymphoma, *ECOG* Eastern Cooperative Oncology Group, *IPI* International Prognostic Index, *SD* standard deviation

### Efficacy

#### Duration of severe neutropenia in cycle 1

Lipegfilgrastim was non-inferior to pegfilgrastim for the reduction in DSN in cycle 1 (Table 2). Mean (standard deviation [SD]) DSN in cycle 1 was 0.8 (0.92) and 0.9 (1.11) days in the two groups, respectively. The adjusted mean DSN difference between groups was − 0.3 days (95% CI, − 0.70 to 0.19). Non-inferiority was also demonstrated in the intent-to-treat population (adjusted mean DSN difference between groups, − 0.1 days [95% CI, − 0.56 to 0.30]).

**Table 2:** Analysis of the primary efficacy endpoint, duration of severe neutropenia in cycle 1

Duration of severe neutropenia (days)	Lipegfilgrastim	Pegfilgrastim
Per-protocol population		
*n*	41	44
Mean (SD)	0.8 (0.92)	0.9 (1.11)
Adjusted mean (95% CI)	0.7 (0.34, 1.11)	1.0 (0.60, 1.36)
Adjusted mean difference between groups (95% CI)	− 0.3 (− 0.70, 0.19)	
Intent-to-treat population*		
*n*	46	50
Mean (SD)	0.8 (0.96)	0.9 (1.08)
Adjusted mean (95% CI)	0.9 (0.29, 1.48)	1.0 (0.44, 1.59)
Adjusted mean difference between groups (95% CI)	− 0.1 (− 0.56, 0.30)	

*CI* confidence interval, *SD* standard deviation

*Patients from the intent-to-treat population with evaluable values for duration of severe neutropenia

#### Incidence of febrile neutropenia and severe neutropenia

Over all cycles, FN according to the strict definition was reported in 1 (2%) patient in each treatment group (during cycle 1 in the lipegfilgrastim group and cycle 6 in the pegfilgrastim group). When assessed using the non-strict definition, FN was reported by 5 (12%) and 2 (5%) patients, respectively.

Very severe neutropenia (ANC < 0.1 × 10^9^/L) in cycle 1 was reported by 5 (12%) patients in the lipegfilgrastim group and 8 (18%) patients in the pegfilgrastim group (adjusted odds ratio, 0.51 [95% CI, 0.131 to 2.016]). Severe neutropenia (ANC < 0.5 × 10^9^/L) was reported by 21 (51%) and 23 (52%) patients, respectively (adjusted odds ratio, 0.99 [95% CI, 0.396 to 2.470]).

#### Absolute neutrophil counts

**Fig. 2:**
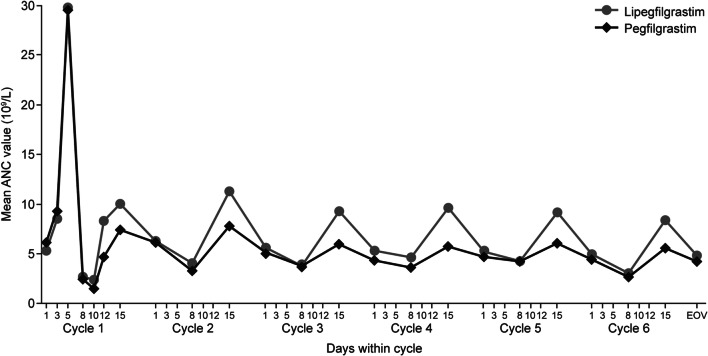
Mean absolute neutrophil count in cycles 1 to 6 by treatment group (per-protocol population). ANC absolute neutrophil count, EOV end-of-treatment visit

Mean daily ANC during cycle 1 is shown in Supplementary Table S2; mean ANC across all cycles is shown in Fig. 2. In both groups, the highest ANC was observed on day 5 of cycle 1. During subsequent cycles, mean ANC peaked on day 15, and was consistently higher in the lipegfilgrastim group. In cycle 1, the ANC nadir was reached around day 10 and was similar in both groups (1.00 ± 1.36 × 10^9^L and 1.19 ± 1.92 × 10^9^L, respectively). The mean time to ANC recovery (≥ 2.0 × 10^9^/L) was 8.3 ± 3.30 days in the lipegfilgrastim group and 9.4 ± 4.92 days in the pegfilgrastim group (Table 3).

**Table 3:** Time to absolute neutrophil count recovery in cycle 1 from the start of chemotherapy (per-protocol population)

ANC recovery criteria	Days to ANC recovery in cycle 1	Lipegfilgrastim (*N* = 41)	Pegfilgrastim (*N* = 44)
≥ 1.0 × 10^9^L	Mean (SD)	6.2 (4.34)	6.8 (5.41)
	Median (range)	8.0 (0–10)	9.5 (0–22)
≥ 1.5 × 10^9^L	Mean (SD)	7.7 (3.69)	8.2 (4.98)
	Median (range)	9.0 (0–12)	10.0 (0–22)
≥ 2.0 × 10^9^L	Mean (SD)	8.3 (3.30)	9.4 (4.92)
	Median (range)	10.0 (0–12)	10.0 (0–22)

*ANC* absolute neutrophil count, *SD* standard deviation

#### Incidence and severity of infection

Over all cycles, infection was reported in 16 (39%) patients in the lipegfilgrastim group and 6 (14%) patients in the pegfilgrastim group (adjusted odds ratio, 4.61 [95% CI, 1.43 to 14.89]). Most infections occurred during cycle 1 (6/16 and 4/6 patients in the two groups, respectively). There was only one infection of grade 4 severity during the study (sepsis in the pegfilgrastim group). Infections of any severity reported by more than a single patient in either group were viral upper respiratory tract infection, herpes zoster, bronchitis, conjunctivitis, oral candidiasis, urinary tract infection, infection, cystitis, influenza, and fungal infection. Infection was microbiologically documented in 4/16 and 2/6 patients in the two groups, respectively.

#### Incidence of hospitalization and antibiotic administration due to febrile neutropenia

Hospitalization due to FN was reported in 5 (12%) patients in the lipegfilgrastim group and 1 (2%) patient in the pegfilgrastim group (all during cycle 1). Intravenous or oral antibiotics were prescribed as treatment or prophylaxis for FN in 16 (39%) and 4 (9%) patients in the two groups, respectively.

#### Chemotherapy dose and delivery

Across all cycles, the median cumulative percentage of the scheduled chemotherapy dose actually delivered was 100% for all drugs in both groups, except for vincristine in the pegfilgrastim group (71.4%). Chemotherapy was administered as planned to all patients in cycle 1. Over cycles 2 to 6, 32 (80%) patients in the lipegfilgrastim group and 33 (75%) in the pegfilgrastim group had delays in their chemotherapy treatment. Two (5%) patients in the pegfilgrastim group omitted at least one chemotherapy cycle. The overall incidence of chemotherapy dose reduction was low in both groups.

### Safety

Almost all patients (98%) reported at least one AE (Supplementary Table S3). The only AE occurring more frequently (≥ 10% difference between groups) in patients receiving lipegfilgrastim was cough. AEs occurring more frequently in patients receiving pegfilgrastim were anemia, nausea, diarrhea, and weight decrease. Bone pain was reported in 2 (4%) patients in the lipegfilgrastim group and 3 (6%) in the pegfilgrastim group. AEs were considered at least possibly related to treatment in 11 (24%) and 10 (20%) patients in the two groups, respectively. No treatment-related AE was reported by more than two patients in either group.

Serious AEs occurred in 46% of patients (21/46 in the lipegfilgrastim group and 23/50 in the pegfilgrastim group). Ten patients withdrew from the study due to AEs (1 [2%] in the lipegfilgrastim group and 9 [18%] in the pegfilgrastim group), none of which were considered treatment-related. Eight patients died during the study (2 in the lipegfilgrastim group, 5 in the pegfilgrastim group, and 1 patient randomized to lipegfilgrastim who died before starting study treatment). No deaths occurred during the treatment period, all were considered to be related to the underlying disease, and none were due to infection.

### Quality of life

There were no noteworthy differences between groups or changes over time for any of the EORTC QLQ-C30 or FACT-N scores over the study period.

## Discussion

This study demonstrated non-inferiority of lipegfilgrastim compared with pegfilgrastim for the reduction of DSN in elderly patients with aggressive B cell NHL receiving myelosuppressive chemotherapy. Results of the analysis in the intent-to-treat population were similar to those in the per-protocol population, confirming the robustness of this finding. Lipegfilgrastim also demonstrated generally comparable efficacy to pegfilgrastim across all secondary endpoints. The incidence of FN was low in both treatment groups, and there were no clinically relevant differences in the incidence of severe or very severe neutropenia, incidence of DSN by duration, depth and time of the ANC nadir, delays in chemotherapy administration, or any quality-of-life measures. Of note, the median cumulative percentage of the scheduled chemotherapy dose actually delivered was 100% for all drugs in both groups, with the exception of vincristine in the pegfilgrastim group. To date, few other studies have assessed the clinical utility of long-acting G-CSFs in this specific patient population [22].

The risk of developing FN in patients receiving R-CHOP21 without G-CSF prophylaxis according to the strict definition used in this study is estimated to be 10–20% [2, 3, 9]. The corresponding incidence of FN in this study was 2% in both groups. This represents a reduction in the incidence of FN of approximately 90%, highlighting the value of treatment with lipegfilgrastim or pegfilgrastim in this patient population. During the study, six patients were hospitalized due to investigator-defined FN (5 in the lipegfilgrastim group and 1 in the pegfilgrastim group). These hospitalizations did not result in an increase in morbidity and mortality, and it could be that other safety issues influenced the need for hospital admission in this high-risk population of elderly cancer patients. The incidence of infection was somewhat higher in the lipegfilgrastim group than in the pegfilgrastim group, but this did not appear to correlate with low neutrophil counts, compromise chemotherapy treatment or lead to increased AEs or serious AEs. Infections were predominantly of grade 3 severity or lower, and the only grade 4 infection occurred in the pegfilgrastim group.

The safety profile of lipegfilgrastim was similar to that of pegfilgrastim, and no new safety signals for lipegfilgrastim were identified. The incidence of AEs was as expected in a population of elderly patients with NHL receiving myelosuppressive chemotherapy, and reported AEs were consistent with the underlying disease and the chemotherapy regimen administered. Bone pain was reported by few patients. More patients withdrew from the study due to AEs in the pegfilgrastim group than in the lipegfilgrastim group; however, none of the AEs leading to withdrawal were considered treatment-related. None of the 8 deaths during the study occurred during the active treatment phase, and all were considered to be related to the underlying disease.

Results of this study are in line with those of a subanalysis of the prospective, non-interventional NADIR study undertaken to evaluate the effectiveness and safety of lipegfilgrastim in patients with NHL undergoing chemotherapy in routine practice settings [26]. A meta-analysis and indirect treatment comparison of lipegfilgrastim versus pegfilgrastim and filgrastim for the reduction of chemotherapy-induced neutropenia and related events demonstrated significant and clinically meaningful differences in favor of lipegfilgrastim for both time to ANC recovery (which is typically longer than DSN) and risk of severe neutropenia [27]. Lipegfilgrastim was also associated with a lower risk of FN over all cycles; however, differences between groups were not statistically significant [27]. As a result of its novel pegylation method [13, 14], lipegfilgrastim has a different pharmacokinetic and pharmacodynamic profile than pegfilgrastim, specifically higher cumulative exposure and slower clearance, and induces a longer-lasting increase in ANC at equivalent doses [15]. Analyses utilizing data from patients with breast cancer suggest that lipegfilgrastim is likely to be a cost-effective alternative to pegfilgrastim for primary prohylaxis of complications of chemotherapy-induced neutropenia and associated complications [28, 29].

In conclusion, this study shows that lipegfilgrastim is an effective option to reduce the duration of severe chemotherapy-induced neutropenia and to prevent febrile neutropenia in elderly patients with aggressive B cell NHL receiving myelosuppressive chemotherapy.

## Electronic supplementary material

ESM 1 (PDF 237 kb)

## Data Availability

The authors confirm to have full control of all primary data. Qualified researchers may request access to patient level data and related study documents including the study protocol and the statistical analysis plan. Requests will be reviewed for scientific merit, product approval status, and conflicts of interest. Patient level data will be de-identified and study documents will be redacted to protect the privacy of trial participants and to protect commercially confidential information. Please email USMedInfo@tevapharm.com to make your request.
