# Supplementary material for: Efficacy and safety of lipegfilgrastim versus pegfilgrastim in elderly patients with aggressive B cell non-Hodgkin lymphoma (B-NHL): results of the randomized, open-label, non-inferiority AVOID neutropenia study
Source: Support Care Cancer. 2020 Sep 17;29(5):2519–27. doi: 10.1007/s00520-020-05711-7 (PMC7981320; doi:10.1007/s00520-020-05711-7)
Supplement: Supplementary file 1 — (PDF 237 kb) [file 520_2020_5711_MOESM1_ESM.pdf]

Supportive Care in Cancer

Efficacy and safety of lipegfilgrastim versus pegfilgrastim in elderly patients with aggressive B-cell non-Hodgkin lymphoma (B-NHL): Results of the randomized, open label, non-inferiority AVOID neutropenia study neutropenia study

Article Type: Original Article

Corresponding Author: Hartmut Link, Prof. Dr.

Westpfalz-Klinikum, Klinik für Innere Medizin

Kaiserslautern, GERMANY

E-Mail: hlink@kabelmail.de

Supplementary Table S1. Overview of key study inclusion and exclusion criteria

**Inclusion criteria**	Age 65–85 years<br>Histological documentation of aggressive B-cell NHL<br>Planned to receive systemic anticancer therapy with at least 6 cycles of R-CHOP21, according to local standards<br>Eastern Cooperative Oncology Group [ECOG] performance status ≤2<br>Life expectancy ≥3 months<br>Adequate bone marrow, renal, and hepatic function as evidenced by the following within 14 days before start of chemotherapy:<br>• Absolute neutrophil count ≥1.5 x 10^9/L<br>• Platelets ≥100 x 10^9/L<br>• Hemoglobin ≥9.0 g/dL<br>• Serum creatinine ≤1.5 x upper limit of the normal range (ULN) OR glomerular filtration rate ≥30 mL/minute/1.73 m^2<br>• Aspartate aminotransferase (AST) and alanine aminotransferase (ALT) ≤2.5 x ULN<br>• Bilirubin ≤1.5 x ULN<br>• Alkaline phosphatase ≤2.5 x ULN
**Exclusion criteria**	Participation in a clinical study ≤30 days before randomization<br>Chemotherapy within the past 3 months (a pre-phase to reduce tumor burden prior to start of R-CHOP was allowed)<br>Any major surgical procedure, open biopsy, or significant traumatic injury within 28 days of start of chemotherapy<br>Active cardiac disease or uncontrolled hypertension<br>Arterial or venous thrombotic or embolic events such as cerebrovascular accident (including transient ischemic attacks), deep vein thrombosis or pulmonary embolism within 6 months before start of chemotherapy<br>Ongoing infection or known history of human immunodeficiency virus (HIV) infection, tuberculosis, or chronic hepatitis B or C<br>Evidence or history of bleeding diathesis<br>Non-healing wound, ulcer, or bone fracture<br>Renal failure requiring hemodialysis or peritoneal dialysis<br>Treatment with lithium at screening or planned during the study<br>Any other conditions that could interfere with study participation or evaluation of the study results

Supplementary Table S2. Summary of observed absolute neutrophil count (x10^9/L) during cycle 1 (per-protocol population)

Absolute neutrophil count (x10^9/L)	Lipegfilgrastim (N=41)	Pegfilgrastim (N=44)
Baseline, n	41	44
Mean (SD)	8.0 (2.55)	8.1 (2.67)
Median (range)	8.2 (2.2–14.7)	7.8 (3.2–13.8)
Day 1, n	34	35
Mean (SD)	5.4 (2.43)	6.1 (2.49)
Median (range)	5.2 (1.2–13.5)	5.6 (2.1–13.1)
Day 3, n	40	40
Mean (SD)	8.6 (2.43)	9.2 (5.75)
Median (range)	8.5 (2.2–14.7)	8.8 (3.2–40.7)
Day 5, n	40	44
Mean (SD)	29.8 (11.20)	29.6 (10.39)
Median (range)	28.6 (5.7–64.6)	30.1 (8.7–52.1)
Day 8, n	41	42
Mean (SD)	2.6 (5.30)	2.6 (3.40)
Median (range)	1.0 (0.1–32.7)	0.9 (0.1–14.9)
Day 10, n	38	42
Mean (SD)	2.3 (2.51)	1.5 (2.27)
Median (range)	1.3 (0.1–9.5)	0.9 (0.0–11.5)
Day 12, n	39	43
Mean (SD)	8.4 (6.36)	4.8 (3.45)
Median (range)	6.3 (1.4–32.6)	4.0 (0.0–15.9)
Day 15, n	40	43
Mean (SD)	10.1 (4.47)	7.5 (3.95)
Median (range)	9.6 (2.5–22.9)	6.5 (1.7–18.1)

SD, standard deviation

Supplementary Table S3. Overview of adverse events (safety population)

**Number of patients, *n* (%)**	**Lipegfilgrastim (N=46)**	**Pegfilgrastim (N=50)**	**Total (N=96)**
At least 1 AE	45 (98)	49 (98)	94 (98)
Individual AEs reported by ≥30% of subjects in either group			
Constipation	18 (39)	16 (32)	34 (35)
Anemia	13 (28)	20 (40)	33 (34)
Neutropenia	17 (37)	15 (30)	32 (33)
Alopecia	16 (35)	13 (26)	29 (30)
Nausea	10 (22)	19 (38)	29 (30)
Fatigue	12 (26)	16 (32)	28 (29)
Diarrhea	9 (20)	16 (32)	25 (26)
At least 1 treatment-related AE	11 (24)	10 (20)	21 (22)
At least 1 SAE	21 (46)	23 (46)	44 (46)
At least 1 treatment-related SAE	0	0	0
At least 1 AE leading to withdrawal from the study	1 (2)	9 (18)	10 (10)
At least 1 AE leading to death*	2 (4)	5 (10)	7 (7)

AE, adverse event; SAE, serious adverse event.
*Causes of death were general physical health deterioration (1 patient) and NHL (1 patient) in the lipegfilgrastim group, and NHL (3 patients), diffuse large B-cell lymphoma (1 patient) and coma (1 patient) in the pegfilgrastim group. One patient randomized to lipegfilgrastim died before starting study treatment.
